# Investigating smiling asymmetries in Parkinson’s disease through the whistle–smile reflex

**DOI:** 10.1007/s00702-026-03193-1

**Published:** 2026-07-02

**Authors:** Adriana Martini, Giulia Paparella, Simone Aloisio, Luca Angelini, Anna Sofia Grandolfo, Martina De Riggi, Daniele Birreci, Davide Costa, Luca Marsili, Matteo Bologna

**Affiliations:** 1 Department of Human Neurosciences, Sapienza University of Rome, Rome, Italy; 2 IRCCS Neuromed, Pozzilli (IS), Italy; 3 Neurophysiopathology Unit, Department of Translational Biomedicine and Neuroscience (DiBraiN), University of Bari Aldo Moro, Bari, Italy; 4 James J. and Joan A. Gardner Center for Parkinson’s disease and Movement disorders, Department of Neurology, University of Cincinnati, Cincinnati, OH USA

**Keywords:** Parkinson’s disease, Hypomimia, Spontaneous smiling, Whistle–smile reflex, Emotional expressivity

## Abstract

**Background:**

Hypomimia is a hallmark of Parkinson’s disease (PD), but whether emotional expressions show lateralized abnormalities remains unclear. While asymmetry has been described for posed smiles, no study has examined true spontaneous smile asymmetry in PD. The whistle–smile reflex (WSR), an involuntary smile elicited after voluntary whistling, offers an opportunity to investigate a form of smiling that closely resembles typical spontaneous expressions of joy. In the present study, we aimed to assess the frequency, intensity, and asymmetry of smiling elicited by the WSR in PD compared with healthy controls (HCs) and to explore links between facial and motor asymmetry. Fifty PD patients and 22 age-matched HCs underwent standardized video recording of the WSR. Only participants judged as smiling by ≥ 3/4 raters were included in the chimeric-face analysis. Left–left (LL) and right–right (RR) chimeric images were generated, and seven blinded raters evaluated smile presence and hemiface expressivity. Facial Laterality Index (FLI) and Body Laterality Index (BLI) quantified asymmetries. WSR was less frequent in PD than HCs (video: 44% vs. 72%; mouth-only chimeras: 50% vs. 81%, *p* < 0.01). PD smiles were more often symmetrical (59% vs. 25%, *p* = 0.03), with no consistent left–right dominance. Facial and motor asymmetry were unrelated (*p* > 0.05). Symmetric smilers had higher UPDRS-III scores (*p* = 0.04). In PD, WSR is reduced and bilaterally flattened, with no lateralized asymmetry, possibly reflecting limbic–subcortical dysfunction. Smile symmetry increases with motor severity, supporting its potential as a simple marker of hypomimia. The WSR and chimeric-smile analysis provide practical tools to assess emotional expressivity in PD.

**Supplementary Information:**

The online version contains supplementary material available at https://doi.org/10.1007/s00702-026-03193-1.

## Introduction

Hypomimia, also known as “facial bradykinesia”, is one of the most characteristic motor symptoms of Parkinson’s disease (PD) (Bologna et al. 2013). It is characterized by reduced and slowed mobility of facial muscles and, consequently, diminished spontaneous and emotional expressiveness, resulting in the typical “masked” appearance that affects patients’ communication (Bologna et al. 2013, Gunnery et al. 2016). Clinically, hypomimia manifests through decreased spontaneous blinking, reduced eyebrow and eyelid motion, and attenuated lower facial expressivity, such as grimacing and smiling (Bologna et al. 2013). Although hypomimia usually appears symmetrical between the two hemifaces, a minority of patients, approximately 5%, exhibit hemihypomimia, a condition marked by unilateral reduction in facial expressivity, which tends to occur ipsilaterally to the most affected body side (Bologna et al. 2013, Ozekmekçi et al. 2007). Ricciardi et al. (2018) investigated the asymmetry of posed facial expressivity in PD using chimeric face techniques (Ricciardi et al. 2018). They found no evidence of consistent left- or right-sided dominance of emotional expression in either PD patients or healthy controls (HCs). However, in PD patients, the most expressive hemiface was significantly correlated with the less affected side of the body, suggesting that facial asymmetry may reflect general motor symptoms’ lateralization rather than hemisphere-specific emotional processing (Ricciardi et al. 2018). This finding parallels the asymmetric onset of PD motor symptoms and raises critical questions about the potential lateralization of facial motor control and its relationship with hemispheric dominance in emotional facial expression, a matter of longstanding debate in the literature (Ricciardi et al. 2018, Marsili et al. 2019, Sedda et al. 2013, Gainotti 1983).

Physiologically, facial expressivity is regulated by a complex integration of motor and limbic circuits, involving cortical areas, particularly the motor and premotor cortices, modulated by subcortical basal ganglia structures, which are closely linked to the dopaminergic system (Ricciardi et al. 2020). Further insight into the physiology of facial expressions has come from the investigation of voluntary vs. spontaneous facial movements (Holstege 1992, Holstege 2002, Wild et al. 2003, Paradiso et al. 2005). While the former primarily engage projections from the primary motor cortex (M1), which particularly targets the contralateral lower facial muscles (Paradiso et al. 2005), the latter are thought to involve a distributed neural network encompassing the left temporo-occipito-parietal junction and lateral prefrontal and basal temporal areas, coordinated by brainstem and subcortical structures, such as the amygdala and basal ganglia (Bologna et al. 2013, Marsili et al. 2019, Ricciardi et al. 2020, Wild et al. 2003, Paradiso et al. 2005, Cruccu et al. 1990). The delineation of these dual systems, voluntary versus emotional facial control, has been supported by lesion studies, demonstrating that lesions involving the corticospinal tracts or M1 typically result in volitional facial paresis, with preserved spontaneous expressions (Bologna et al. 2013, Holstege 2002, Hopf et al. 1992), whereas damage to limbic or subcortical areas can selectively impair emotional expression, a phenomenon known as emotional facial paresis (Wild et al. 2003, Morecraft et al. 2004). This dissociation is clinically relevant in PD, where patients frequently retain the ability to produce voluntary facial movements while exhibiting a reduced or emotionless facial appearance (Bologna et al. 2013, Wild et al. 2003, Hopf et al. 1992). In this regard, recent clinical, neuroimaging, and neurophysiological studies in PD have demonstrated that hypomimia is linked to both central dopaminergic deficits, sharing common pathophysiological mechanisms with other motor symptoms (Bologna et al. 2013, Ricciardi et al. 2020), and to alterations of a broader network, including the medial frontal cortex, particularly the cingulate cortex (area M4), which mediates voluntary facial movements with an emotional component, as well as the insula and other limbic regions, responsible for emotion processing and expression (Bologna et al. 2013, Ricciardi et al. 2018, Ricciardi et al. 2020, Morecraft et al. 2004, Ricciardi et al. 2015, Gothard 2014).

In this framework, the act of smiling holds relevance. It may arise spontaneously (e.g., in response to humor or social stimuli) or be generated voluntarily (e.g., posed) upon instruction (Wild et al. 2003). Spontaneous smiles are characterized by activation of both the zygomatic major and orbicularis oculi muscles, resulting in the lifting of the lip corners and characteristic eye crinkling (Wild et al. 2003, Ekman et al. 1990). Conversely, voluntary smiling may lack emotional depth and is often associated with asymmetric or incomplete lower face activation (Marsili et al. 2019). From a neurophysiological perspective, spontaneous smiling is thought to involve medial cortical areas involved in emotion, including the insula, and subcortical structures, such as the amygdala and the striatocapsular region, while posed (voluntary) smiling is mainly mediated by cortical motor areas and the cingulate cortex, which integrates limbic influences and differentiates smiling from other voluntary facial movements. (Holstege 2002, Hopf et al. 1992, Marsili et al. 2014).

Within this context, the whistle-smile reflex (WSR) has emerged as a potentially valuable clinical sign (Paparella et al. 2024, Hanes 1943, Monteiro et al. 2022). It is defined as the involuntary smile that follows the voluntary act of whistling in response to the physician’s request, which may appear incongruous during a medical examination. In healthy individuals, this reflexive smile commonly occurs and is thought to reflect the integrity of emotional–motor integration pathways. In contrast, the WSR is frequently absent in PD patients, possibly suggesting disruptions within the neural circuitry that links volitional and spontaneous/emotional facial movements. We recently demonstrated that almost 80% of patients with PD lack the WSR, and that its absence correlates with hypomimia rather than with emotional impairment (Paparella et al. 2024). These results reinforce the hypothesis that WSR could act as a simple yet sensitive clinical probe of facial bradykinesia (Paparella et al. 2024).

In this study, we hypothesized that WSR asymmetry could provide useful information regarding the pathophysiology of emotional facial expression motor control in PD. Indeed, in PD, both posed smiling and voluntary grinning can be impaired (Marsili et al. 2014). However, while there is previous evidence of facial asymmetry during posed smiling in PD, (Ricciardi et al. 2018) no research has examined potential asymmetries in a form of smiling that closely resembles typical spontaneous expressions. In this context, the WSR offers a compelling opportunity to investigate such phenomena (Paparella et al. 2024). We investigated the asymmetry of the WSR in PD patients compared with HCs. The smilers were identified through a multi-rater evaluation, and chimeric images generated from their video recordings were assessed to quantify facial symmetry. Within the PD group, we further examined the relationship between facial and body motor asymmetry, as well as demographic and clinical differences between symmetric and asymmetric smiling patterns.

## Methods

### Participants and clinical assessment

We consecutively enrolled 50 PD patients (35 males; mean age: 70.8 ± 9.9 years), diagnosed according to the most recent clinical diagnostic criteria (Postuma et al. 2015), and 22 HCs (13 males; mean age: 71.6 ± 8.4 years). The study was conducted at the Department of Human Neurosciences, Sapienza University of Rome, and at the Gardner Family Center for Parkinson’s Disease and Movement Disorders, Department of Neurology, University of Cincinnati, Cincinnati, OH, USA, from January 2023 to September 2025. The exclusion criteria included atypical or secondary parkinsonism, dementia, psychiatric disorders unrelated to PD, other neurological or neuromuscular conditions affecting facial expressivity, severe visual or auditory impairments, recent facial surgery or trauma, and substance abuse. Participants underwent a single experimental session, and all patient evaluations were conducted during the ON medication state, defined as the period of optimal motor benefit occurring approximately 60–90 min after the usual dose of dopaminergic medication. For each participant, a comprehensive clinical assessment was performed, including demographic and medical history collection and a detailed neurological examination. Motor symptoms were evaluated using the Movement Disorder Society–sponsored Unified Parkinson’s Disease Rating Scale (Goetz et al. 2008, Antonini et al. 2013), part III (MDS-UPDRS III); non-motor symptoms with the Non-Motor Symptoms Scale for Parkinson’s Disease (NMSS) (Wamelen et al. 2021); cognitive function with the Montreal Cognitive Assessment (MoCA) (Nasreddine et al. 2005); and psychiatric comorbidities with the Beck Anxiety Inventory (BAI) (Beck et al. 1988) and the Beck Depression Inventory-I (BDI) (Beck et al. 1961). In patients, the predominant side of motor symptom onset was determined, and dopaminergic therapy was expressed as Levodopa Equivalent Daily Dose (LEDD) (Jost et al. 2023). The study was approved by the local ethics committee, adhered to the principles of the Declaration of Helsinki, and written informed consent was obtained from all participants.

### Video recordings and evaluation

All subjects underwent a standardized video recording (Ricciardi et al. 2015, Paparella et al. 2024, Ricciardi et al. 2017). Participants were seated without head support, feet resting on the floor, and hands at rest. Participants’ faces were recorded in a relaxed state for 30 seconds while looking at the camera (“spontaneous facial expression” videos). Using a standard phrase, participants were then asked to perform a brief whistle, and the recording continued for the next 15 seconds. The recording encompassed the upper body, including the head, hair, and shoulders, with a high-resolution camera situated approximately 60 cm from the subject. Offline video editing was performed by cropping the upper part of the face and shoulders, leaving only the mouth area visible to ensure blinded assessment. Videos were randomized and presented to 4 experienced movement disorders specialists. During the evaluation of the “whistle-smile reflex” videos, raters indicated with a binary code (0: no; 1: yes) whether they observed an immediate smile after whistling. After each choice, the raters were also asked to grade their confidence level in the accuracy of their response, from 0 (“no confidence at all”), to 10 (“maximum confidence”).

### Chimeric pictures and evaluation

For the subsequent analysis, we selected only videos of participants (PD patients and HCs) who had smiled after whistling according to at least 3 out of 4 raters. For each selected video, we first extracted the single frame showing the most pronounced smile to obtain a static image, and then we generated a pair of chimeric images of each original smile, by combining the left-left (LL) and right-right (RR) half of the faces from the original picture, using the software Pichacks (http://www.pichacks.com), as in previous studies (Ricciardi et al. 2018, Marsili et al. 2019). Specifically, for each picture, the original image was divided vertically through the midline, and the two LL and two RR hemifaces were combined to make two chimeric faces (whole-face versions). Each chimeric image obtained was then further processed to generate mouth-only chimeric versions for each participant. Therefore, we presented these chimeric pictures to seven raters, who were blind to the purpose of the study and were different from those who had previously assessed the WSR. A total of 88 images were generated for PD patients and 64 for HCs, of which 44 and 32, respectively, depicted only the mouth. These images were presented first to the raters, followed by an equal number of images showing the entire face. Images were shown to raters one pair at a time, side by side on a computer screen, with left–right positions randomized across trials, to rule out any spatial attention bias. Raters were asked to evaluate whether each image depicted a smile, using a binary code (yes/no). We collected their responses along with their confidence levels, ranging from 0 (no confidence) to 10 (maximum confidence). Additionally, raters were instructed to assess which of the two chimeric images appeared more expressive. Specifically, they were asked to judge whether the two chimeric images of each patient were comparable in terms of expressiveness (i.e., whether both were smiling or not smiling to a similar extent). In this task, raters could respond with 0, if they considered the two images comparable, -1 if they judged the image on the left side of the computer screen to be more expressive and + 1 if they judged the image on the right side to be more expressive.

### Chimeric images rating analysis

**Fig. 1:**
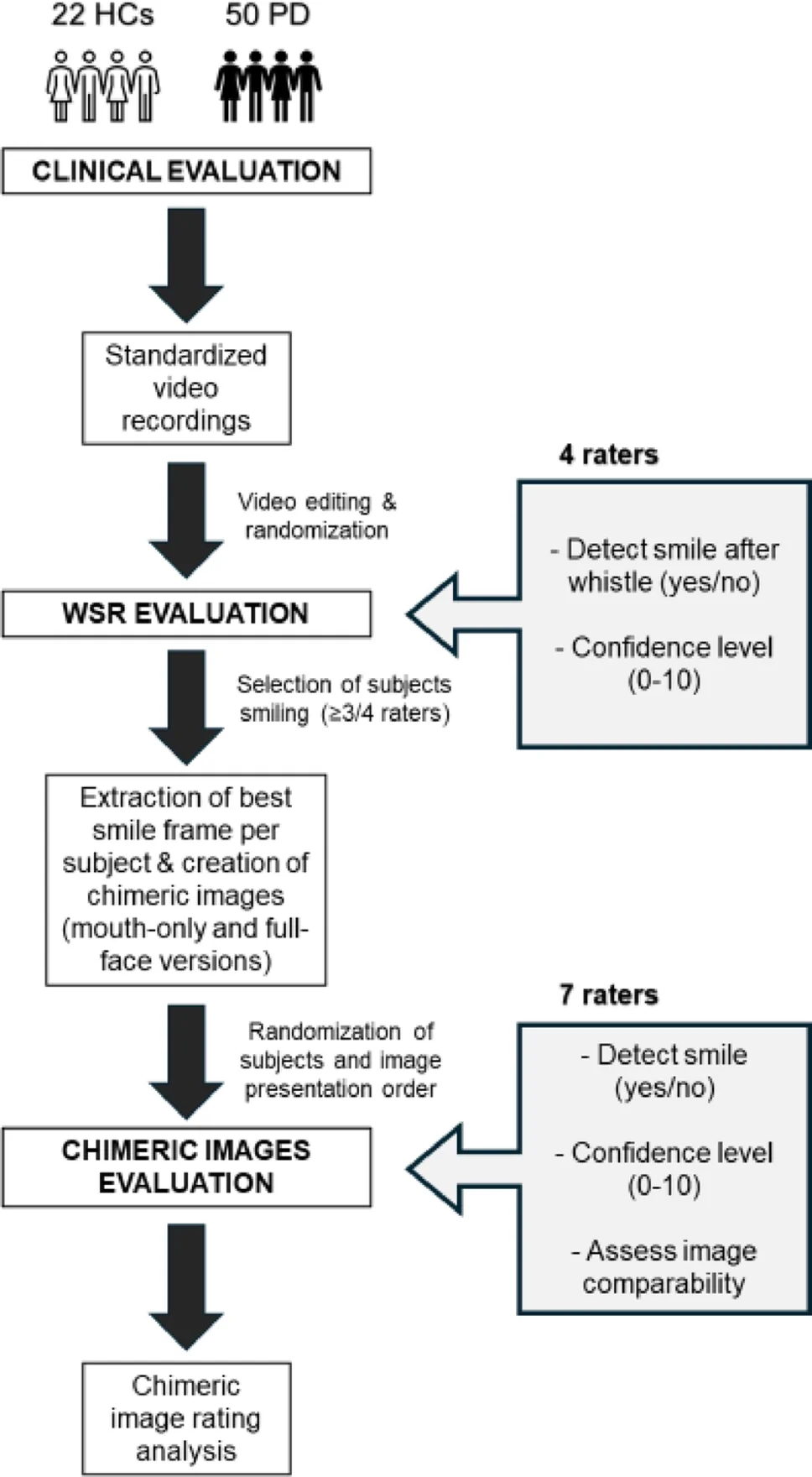
Schematic representation of the experimental design. *PD*  Parkinson’s Disease, *HCs*  Healthy Controls, *WSR*  Whistle-Smile Reflex

Experimental design is illustrated in Fig. 1. From the raters’ evaluations, we first counted the images judged as showing a smile and the number of subjects whose two chimeric images were rated as comparable, both defined according to ratings provided by at least 4 out of 7 evaluators, and then we compared these results between PD patients and HCs.

To investigate whether the hemiface judged as less smiling in each pair of images corresponded to the most affected side of the body, we calculated two indices: the Body Laterality Index (BLI) and the Facial Laterality Index (FLI) (Ricciardi et al. 2018). The BLI was computed by summing the MDS-UPDRS-III items related to bradykinesia, rigidity and tremor in the limbs on the left and right sides. The FLI was derived by summing, for each subject, the number of “smiling” responses received by the left–left and right–right chimeric images across raters. These sums were defined as LL and RR, respectively. Both indices were then calculated using the following formula: (LL − RR) / (LL + RR) × 100, yielding a continuous measure of lateralization for both motor symptoms and facial expressivity. Analyses were conducted separately for chimeric images depicting only the mouth and for those showing the entire face, to assess potential differences related to the extent of facial information provided.

Finally, we divided the PD patients into two subgroups: patients with comparable (and thus symmetric) chimeric images and patients with non-comparable images, based solely on the analysis of mouth-only images. This approach was adopted to avoid confounding factors related to full-face evaluation, and because hypomimia is known to be more pronounced in the lower half of the face as reported in the literature (Ozekmekçi et al. 2007). To investigate the potential presence of a more axial phenotype of the disease in either subgroup, an Axial Score was calculated for each patient by summing the MDS-UPDRS-III items related to speech (item 18), rigidity of the neck (item 22), arising from chair (item 27), posture (item 28), gait (item 29) and postural stability (item 30).

In the HCs group, we evaluated whether there was left or right predominance in smile expression by counting how many times the RR or LL chimeric images were judged as more smiling.

### Statistical analysis

Normality of the data was assessed using the Shapiro–Wilk test. Parametric statistical tests (unpaired t test) were applied only when normality assumptions were met to compared PD patients with HCs; otherwise, we used non-parametric procedures (Mann–Whitney U test). Categorical data in the two groups were compared using Fisher’s exact test. Inter-rater agreement was computed using Fleiss’ κ coefficient (Landis and Koch 1977). Fleiss' κ values were interpreted based on the Landis-Koch classification which defines six levels of reliability: <0.00 poor agreement; 0.01–0.20 slight agreement; 0.21–0.40 fair agreement; 0.41–0.60 moderate agreement; 0.61–0.80 substantial agreement; 0.81–1 almost perfect agreement (Paparella et al. 2024, Landis and Koch 1977).

Parametric and non-parametric tests were also used to compare clinical and demographic variables between patients whose chimeric smiles were judged as symmetric or asymmetric. Spearman’s rank correlation coefficients were calculated to assess potential associations between clinical scores and clinico-demographic measures, as well as between indices of body and facial lateralization, specifically between the most expressive hemiface and the most affected side of the body. Data are presented as median (interquartile range, IQR), unless otherwise specified. Results were considered significant for *p* < 0.05. Effect sizes were calculated for all statistically significant comparisons and expressed as squared measures of association. For categorical variables, φ² was reported. For continuous comparisons, r² was reported, derived from rank-biserial correlations for non-parametric analyses and from Cohen’s d for parametric comparisons. For all statistical procedures, we used Jamovi statistical software (version 2.6.44).

## Results

### PD patients vs. HCs

#### Demographic data and clinical evaluation

PD patients and HCs were similar in terms of age (*p* = 0.76 by unpaired t test) and sex distribution [PD: 35 males (M) (70%); HCs: 13 M (59.1%); *p* = 0.42]. Among patients with PD, the median disease duration was 6 (7.5) years, the total MDS-UPDRS-III score was 25.5 (16), the total NMSS score was 41.5 (46.8) and the mean LEDD was 600 (394). BDI and BAI scores were higher in PD patients than in HCs (both *p* = 0.01, r² = 0.14 for both comparisons). Despite a small-to-moderate strength of association, these findings are consistent with greater anxiety and depressive symptom burden in the patient group. No differences were found between the two groups regarding the MoCA score (*p* = 0.09) (Table 1).

**Table 1:** Demographic and clinical data of Parkinson’s Disease (PD) patients and healthy controls (HCs)

*N*°	PD	HCs	*p*-value
	50	22	/
Age	70.8 ± 9.9	71.6 ± 8.4	0.76
Sex	35 M (70%)	13 M (59.1%)	0.42
Disease Duration	6 (7.5)	/	/
LEDD	600 (394)	/	/
MDS-UPDRS part III	25.5 (16)	/	/
Most affected side	26 Right (52%); 24 Left (48%)	/	/
NMSS	41.5 (46.8)	/	/
MOCA	26 (2.75)	25 (2)	0.09
BDI	5 (8)	0 (5)	**0.01**
BAI	8 (12.8)	0 (7.25)	**0.01**

Age is expressed in years. *M* males, *MoCA* Montreal Cognitive Assessment, *MDS-UPDRS* Movement Disorder Society–sponsored Unified Parkinson’s Disease Rating Scale part III, *NMSS* Non-Motor Symptoms Scale for Parkinson’s Disease, *BDI* Beck Depression Inventory, *BAI* Beck Anxiety Inventory. Data are presented as median (interquartile range, IQR), except for age, which was normally distributed and is reported as mean ± standard deviation (SD). *p*-values were obtained using the unpaired t test for age comparisons, the Mann–Whitney U test for continuous non-normally distributed variables, and Fisher’s exact test for sex. Statistically significant *p* values are shown in bold

### WSR video evaluation

As expected, PD patients were less frequently rated as smiling than HCs after the whistle. Specifically, only 22 out of 50 PD patients (44%) were judged to be smiling by at least 3 out of 4 raters, compared with 16 out of 22 HCs (72%). The inter-rater agreement in the evaluation was substantial for the PD group (Fleiss’ κ = 0.66) and moderate for the HCs group (Fleiss’ κ = 0.54).

Clinical and demographic characteristics were comparable between PD patients who exhibited a WSR and those who did not (Supplementary Table 1), with the exception of age, which was slightly higher in PD non-smilers than in PD smilers (*p* = 0.02 by unpaired t test, r² = 0.11, indicating a small-to-moderate association). Similarly, clinical and demographic variables did not differ between PD patients and HCs who smiled after the whistle (Supplementary Table 2), indicating that these subgroup analyses mirrored the results observed in the overall sample.

#### Chimeric images evaluation

**Fig. 2:**
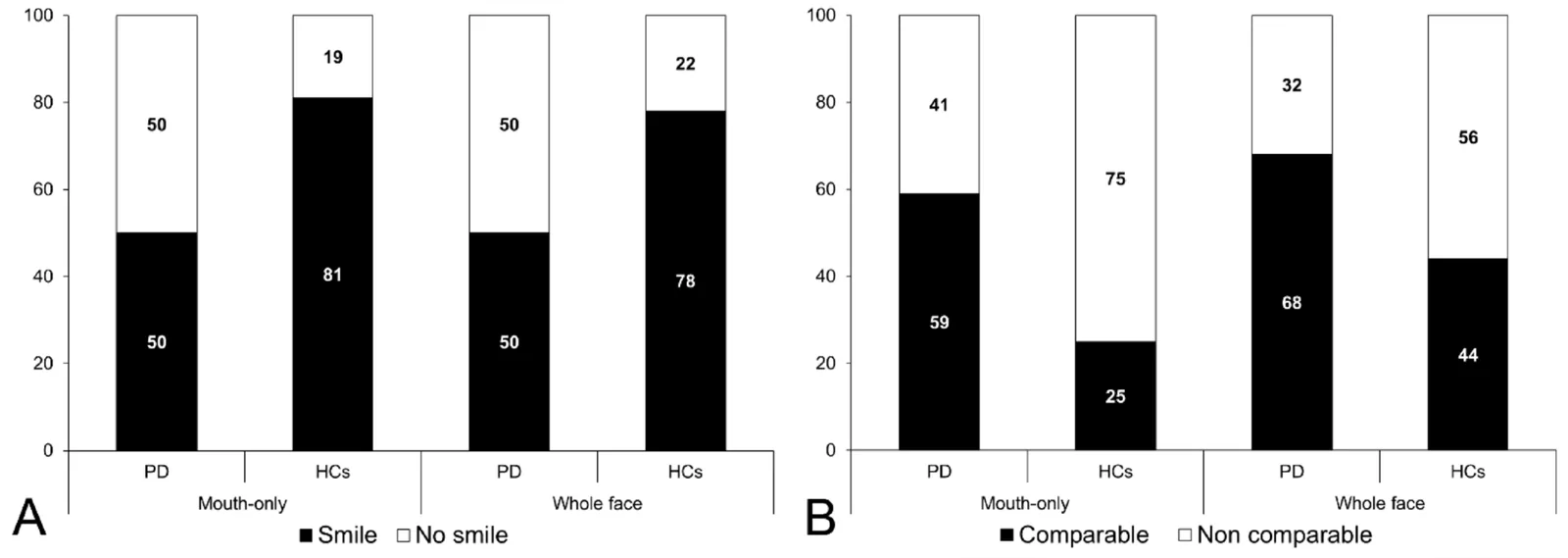
A: Percentages of Parkinson’s disease (PD) patients and healthy controls (HCs) classified as smiling or non-smiling after whistling in the evaluation of mouth-only and whole-face chimeric images. Note that, in both cases, PD patients were significantly less frequently classified as smiling than HCs. B: Percentages of pairs of chimeric images depicting PD patients or HCs that were classified as comparable or non-comparable, for both the mouth-only and whole-face versions. Notably, the two hemifaces appeared more symmetrical in PD patients

In the evaluation of mouth-only chimeric images, PD patients smiled significantly less than HCs. Specifically, only 50% of the chimeric images of PD patients (22 out of 44) were judged as smiling, compared with 81.25% (26 out of 32) among HCs (Fig. 2A) (*p* < 0.01, φ² = 0.10), indicating a relatively modest behavioral difference, with approximately 10% of the variance explained by group membership. The overall inter-rater agreement for smile assessment was substantial in both groups (Fleiss’ κ = 0.67 in PD patients and 0.62 in HCs). Agreement among evaluators in assessing whether the images were comparable was moderate, both in PD patients (Fleiss’ κ = 0.43) and in HCs (Fleiss’ κ = 0.50). The two images were considered comparable in 13 out of 22 PD patients (59.1%), whereas this occurred in only 4 out of 16 HCs (25%) (*p* = 0.03, *φ² = 0.12*) (Fig. 2B), suggesting that the two hemifaces were more symmetrical in PD patients. There were no significant differences in raters’ confidence levels when evaluating the presence of a smile in the two chimeric images between PD and HCs groups (*p* = 0.15).

In the assessment of whole-face chimeric images, 22 out of 44 images (50%) of PD patients and 25 out of 32 images (78.1%) of HCs were judged as smiling (*p* = 0.001, φ² = 0.08). Despite the modest effect size, these results indicated that smiling images were more frequently observed in HCs (Fig. 2A), and were consistent with those obtained from the evaluation of mouth-only chimeric images. Inter-rater agreement for smile assessment in full-face images was moderate in the PD group (Fleiss’ κ = 0.57) and substantial in HCs (Fleiss’ κ = 0.68). Conversely, agreement among evaluators on the comparability of the two images was only fair in both PD patients (Fleiss’ κ = 0.31) and HCs (Fleiss’ κ = 0.31). Moreover, the images were judged as comparable in 15 out of 22 PD patients (68.2%) and in 7 out of 16 HCs (43%) (Fig. 2B), but no significant difference was found between the two groups (*p* = 0.12). Finally, as in the mouth-only condition, no significant differences were found between the PD and HCs groups in raters’ confidence levels when judging the presence of a smile in the full-face chimeric images (*p* = 0.30).

**Fig. 3:**
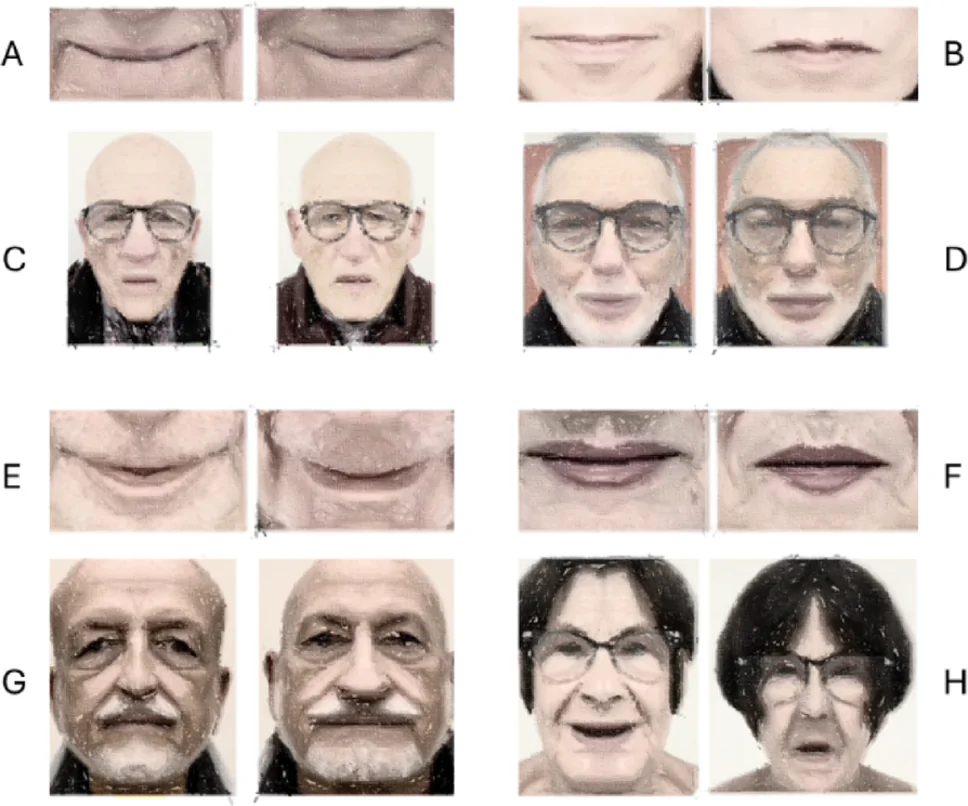
Chimeric images of Parkinson’s disease (PD) patients and healthy controls (HCs), rated for smile comparability. A PD, RR–LL, mouth-only, rated as comparable by all 7 raters. B PD, LL–RR, mouth-only, rated as non-comparable; the right image was judged as showing a more pronounced smile. C PD, RR–LL, whole-face, rated as comparable by all raters. D PD, RR–LL, whole-face, rated as non-comparable by 6/7 raters; right image judged as showing a more pronounced smile. E HC, LL–RR, mouth-only, comparable by 6/7 raters. F HC, RR–LL, mouth-only, non-comparable by 6/7 raters; right image judged as showing a more pronounced smile. G HC, RR–LL, whole- face, comparable by all raters. H HC, LL–RR, whole-face, non-comparable by 6/7 raters; right image judged as showing a more pronounced smile. RR = Right–Right; LL = Left–Left. Note: To preserve participant anonymity, all images have been stylized as sketches, so that facial features are clearly visible but individual identities are not recognizable

In summary, similar results were obtained from the evaluation of chimeric images showing only the mouth and those depicting the whole face, although inter-rater agreement in assessing comparability was lower for whole-face images in both groups. In both conditions, images derived from PD patients were judged as smiling less frequently and more often rated as comparable, indicating reduced facial expressiveness together with greater symmetry in the PD group. Examples of whole-face and mouth-only image pairs considered comparable or non-comparable by at least 4 of 7 raters in both PD patients and HCs are shown in Fig. 3.

### Sub-analysis within the PD group

**Fig. 4:**
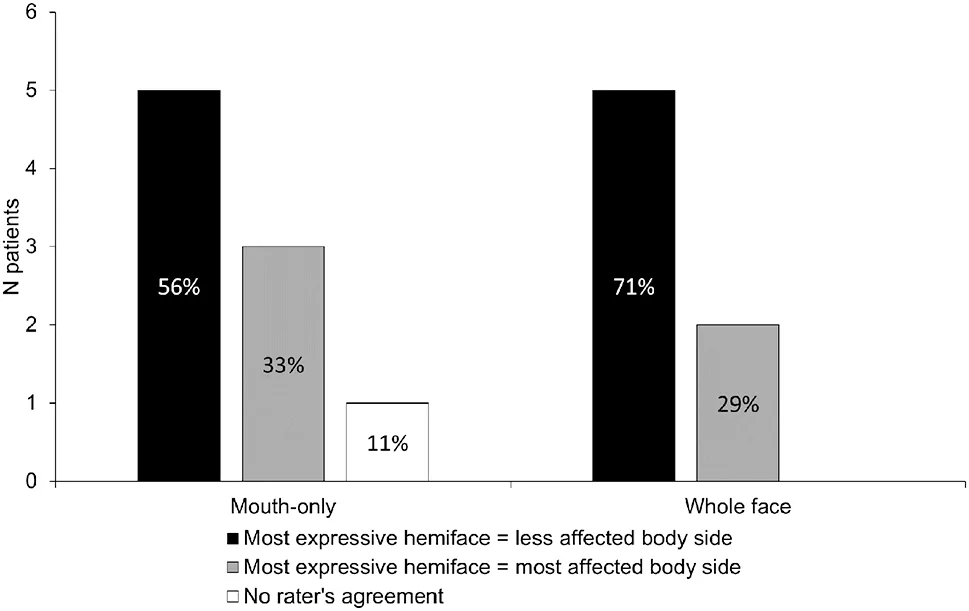
Relationship between facial and body laterality in Parkinson’s disease (PD). Distribution of PD patients with non-comparable chimeric images according to whether the most expressive hemiface corresponded to the less affected or the most affected side of the body. Results are shown separately for mouth-only and whole-face conditions. In PD patients, facial expressions during the whistle–smile reflex (WSR) tended to be slightly more expressive on the less affected side of the body; however, no consistent pattern of facial lateralization was observed

In the PD group, chimeric images showing only the mouth were considered non-comparable in 9 patients. Among these patients, 56% were judged to exhibit a more pronounced smile on the hemiface corresponding to the less affected body side, whereas in 33% the hemiface corresponding to the most affected side appeared more expressive. For one patient (11%), no consensus was reached among raters regarding which chimeric image showed a more pronounced smile or whether the images were comparable (Fig. 4).

Similarly, chimeric images showing the whole face were considered non-comparable in 7 patients. Among these patients, 71% were judged to exhibit a more pronounced smile on the hemiface corresponding to the less affected body side, whereas in 29% the hemiface corresponding to the most affected side appeared more expressive (Fig. 4).

Mean raters’ confidence levels did not differ between comparable and non-comparable whole-face chimeric images (7.91 ± 1.13 vs. 7 ± 0.96, *p* = 0.08), whereas higher confidence levels were observed for mouth-only chimeric images in patients classified as comparable compared to those classified as non-comparable (8.49 ± 0.98 vs. 7.30 ± 0.75, *p* = 0.006, r² = 0.32), indicating a moderate association, with approximately 32% of the variance explained by group membership.

The FLI derived from mouth-only chimeric images was − 33.3 ± 48.7 (mean ± standard deviation), while that obtained from whole-face images was − 21.21 ± 66.74. The BLI was − 0.26 ± 42.2, and the axial score was 8.13 ± 6.62. Only the mouth-based FLI showed a significant difference between the symmetric and asymmetric patient groups (*p* = 0.01, r² = 0.19), with asymmetric patients displaying more dispersed values. No differences emerged between the two PD subgroups regarding the whol-face-based FLI and the axial score (all *p* > 0.05). Moreover, patients with symmetric faces showed higher total MDS-UPDRS-III scores than those with asymmetric faces (*p* = 0.04, r² = 0.29, indicating a moderate association). Conversely, no significant differences were found between the two groups in MoCA (*p* = 0.21), BDI (*p* = 0.66), BAI (*p* = 0.54), or NMSS (*p* = 0.65) scores, indicating that neither cognitive nor psychiatric factors contributed to the observed difference between symmetric and asymmetric patients. No other differences were observed in disease duration, age, LEDD, or sex (all *p* > 0.05). Finally, no correlations emerged between clinical-demographic measures and the laterality indices or axial score (all *p* > 0.05).

### Sub-analysis within the HC group

In the HC group, chimeric images were more frequently judged as asymmetric, although no consistent right- or left-sided predominance in smiling was observed. In the evaluation of mouth-only chimeric images, 12 out of 16 subjects (75%) were rated as having non-comparable images. Among these, 5 (31.3%) were judged as smiling more on the right and 5 (31.3%) on the left, while in 2 subjects (12.5%) no clear consensus was reached for either side. Similarly, in the evaluation of full-face chimeric images, 9 subjects (56.3%) were rated as having non-comparable images; among them, 5 (31.3%) were judged as smiling more on the left and 4 (25%) on the right.

## Discussion

This study examined facial expressivity and asymmetry during the WSR in PD patients compared with HCs, through both direct observation and the analysis of chimeric facial images. Compared with HCs, patients with PD: (1) exhibited a reduced frequency of WSR; (2) showed more symmetrical WSR in the chimeric image evaluations and (3) demonstrated greater facial symmetry in association with higher global motor impairment, whereas cognitive and psychiatric factors showed no significant relationship with facial symmetry.

### PD patients vs. HCs

PD patients were rated as smiling significantly less frequently than HCs across both the WSR video evaluations and the chimeric image assessments. This consistent reduction in WSR is in line with previous evidence of diminished emotional facial expressivity in PD, a hallmark of hypomimia (Bologna et al. 2013, Paparella et al. 2024, Madeley et al. 1995). Prior studies have shown that hypomimia affects both voluntary (“posed”) and spontaneous expressions, leading to decreased amplitude and frequency of facial movements (Jacobs et al. 1995, Smith et al. 1996, Simons et al. 2004, Buck and Duffy 1980, Katsikitis and Pilowsky 1991). In the present study, the WSR likely reflects a blunted spontaneous response and impaired automatic activation of emotional motor pathways, resulting in an overall flattening of facial expressivity.

Regarding facial symmetry, prior studies in healthy individuals have reported hemispheric lateralization of emotional expression (Sedda et al. 2013, Gainotti 1983), meaning that dominance of one hemisphere leads to asymmetry in facial expressivity. Borod et al. (1983) investigated facial asymmetry in posed and spontaneous emotional expressions and demonstrated significant left-sided lateralization, implying right-hemisphere predominance, particularly for negative emotions, without differences between voluntary and spontaneous expressions (Borod et al. 1983). Although theoretical models diverge, with some supporting a general right-hemisphere dominance (Borod et al. 1983, Fernández-Carriba et al. 2002, Gainotti 1972, Gainotti 2012, Gainotti 2021, McLaren and Bryson 1987 ) and others a valence-dependent lateralization, with the left hemisphere mediating positive emotions and the right hemisphere negative ones (Wager et al. 2003, Bruyer 1980, Sackeim et al. 1982, Ahern and Schwartz 1985, Silberman and Weingartner 1986), our HC group displayed greater smiling asymmetry, consistent with this literature. Conversely, in PD patients, this asymmetry was markedly reduced: chimeric images were more often rated as comparable, indicating a more symmetrical facial pattern. This finding likely reflects the bilateral and uniform flattening of facial movement due to hypomimia, which obscures the natural asymmetrical patterns of emotional expression. In support of this interpretation, raters reported higher confidence levels when evaluating mouth-only chimeric images classified as comparable, indicating that increased facial symmetry in PD is unlikely to arise from perceptual ambiguity or rating uncertainty. Rather, it appears to reflect a generalized reduction in the intensity of facial expressivity. PD patients therefore appear to smile less frequently and less intensely, with globally flatter and less differentiated expressions. These observations are consistent with previous reports that hemi-hypomimia is rare in PD (Ozekmekçi et al. 2007; Zingler et al. 2005; Crosiers et al. 2011) and with neurophysiological studies showing no lateralized differences in the kinematics of facial expressions (Bologna et al. 2016) or in posed smile and grinning (Smith et al. 1996).

Raters’ confidence in identifying smiles did not differ between PD and HCs groups, regardless of whether only the mouth or the entire face was evaluated. However, inter-rater agreement was lower for full-face than for mouth-only images, suggesting that evaluating smile symmetry is more complex when the entire face is considered. This difference likely reflects the higher perceptual and emotional complexity of full-face expressions. Spontaneous smiles, such as those elicited by the WSR, are generated through a distributed limbic–subcortical network that produces a coordinated but not necessarily synchronous activation of multiple facial regions (Rinn 1984). In natural emotional expressions, upper- and lower-face components often vary in intensity and timing, and the presence of neutral or weakly expressive ocular or forehead movements can attenuate, distort, or even contradict the signal conveyed by the mouth. This internal heterogeneity makes the overall expression harder to categorize, particularly when facial features convey partially incongruent emotional cues (e.g., a smiling mouth with neutral eyes).

Moreover, from a perceptual standpoint, observers rely more heavily on the lower face when judging the presence and intensity of a smile, as the mouth provides the most salient and unambiguous cue for positive affect. In contrast, the upper face, especially the eyes, contributes more to emotional authenticity (Ambadar et al. 2005, Krumhuber and Kappas 2005) but also introduces greater variability. In PD, this variability may be amplified by hypomimia, which disproportionately reduces upper-face involvement and can lead to expressions that appear flattened or ambiguous.

An additional factor that may have contributed to the lower agreement for full-face images is the intrinsic limitation of using static frames to represent a fundamentally dynamic facial behaviour. Natural smiles unfold over time through characteristic onset, apex, and offset phases, and temporal dynamics provide crucial cues for interpreting emotional authenticity and facial symmetry. These dynamic cues substantially enhance the perception of subtle expressions (Ambadar et al. 2005, Krumhuber and Kappas 2005, Dobs et al. 2018). When reduced to a single static frame, particularly in PD, where facial movements are bradykinetic and diminished in amplitude, many of these diagnostic temporal features are lost, increasing perceptual ambiguity and disproportionately affecting full-face evaluations.

As a result, full-face assessments require integrating multiple subtle features that may differ in both salience and temporal coordination, lowering agreement among raters. Mouth-only images, by isolating the most informative region for smile perception, reduce perceptual noise and ambiguity, thereby increasing inter-rater reliability.

### PD subgroup analysis

The absence of correlation between the FLI and the BLI further supports the notion that facial and bodily motor symptoms in PD are, at least partially, independent. While limb motor symptoms typically exhibit clear lateralization (Wager et al. 2003, Silberman and Weingartner 1986), facial hypomimia seems to evolve bilaterally, suggesting distinct neural mechanisms underlying facial versus limb motor control. This interpretation aligns with previous findings on posed smiling (Marsili et al. 2014) but contrasts with those of Ricciardi et al, who reported an association between emotional expression asymmetry and motor lateralization, suggesting a shared neural substrate. However, it is important to note that Ricciardi et al. assessed emotional asymmetry using posed smiles, a task that relies on corticobulbar voluntary motor pathways and therefore may reflect the same cortical mechanisms that drive motor lateralization in the limbs. In contrast, the present study is the first to investigate asymmetry during a form of smiling that closely resembles typical spontaneous expressions of joy. Because spontaneous smiles are mediated predominantly by limbic–subcortical emotional–motor circuits that bypass the primary motor cortex, they are likely less influenced by the lateralized cortical mechanisms underlying limb motor signs (Bologna et al. 2013, Marsili et al. 2019, Holstege 1992, Wild et al. 2003, Paradiso et al. 2005). This fundamental difference in the neural substrates of posed versus spontaneous facial expressions may therefore explain the absence of correlation between facial laterality and motor asymmetry in our cohort. Comparisons between the present findings and prior reports on posed/voluntary smiles in PD, however, are inherently indirect and may be influenced by differences in task demands, rating methods, and patient characteristics across studies. Moreover, our analysis relied on a small subset of asymmetric cases. Future studies investigating both spontaneous and posed/voluntary smiles in the same sample, including a larger number of patients, are needed to clarify these issues.

Within the PD group, patients whose chimeric images were judged as more symmetric exhibited higher MDS-UPDRS-III scores than those with asymmetric smiles. This may indicate that as the disease progresses and motor dysfunction increases, facial expressivity becomes globally reduced, resulting in apparent facial symmetry. In contrast, patients with milder motor impairment may still display residual asymmetries, resembling the physiological lateralization observed in healthy individuals. These findings are partially consistent with those of Marsili et al., who demonstrated that abnormalities of posed smiling correlate with greater motor dysfunction in PD, (Marsili et al.^2014^) and further support the view that facial asymmetry may characterize the early stages of PD, diminishing as hypomimia becomes more diffuse and bilateral (Ozekmekçi et al. 2007, Zingler et al. 2005, Crosiers et al. 2011), similarly to other motor symptoms as the neurodegeneration advances (Marinus and Hilten 2015).

No relationship emerged between facial symmetry and axial symptoms’ severity, as measured by the axial score. Although hypomimia itself can be considered an axial feature, often being associated with orofacial symptoms and other axial signs (Ricciardi et al. 2020), facial symmetry does not appear to serve as a reliable indicator of axial involvement. However, the discrepancy with previous work may be explained by fundamental methodological and physiological differences. Earlier studies (Ricciardi et al. 2020) evaluated hypomimia using the MDS-UPDRS facial-expression item, which captures global facial bradykinesia across multiple orofacial and axial domains and therefore tends to co-vary with other axial features and with dopaminergic responsiveness. In contrast, our measure isolates a very specific behaviour, the symmetry of the WSR, which possibly reflects the output of limbic–subcortical emotional–motor circuits rather than the broader cortico–brainstem networks typically implicated in axial dysfunction (Bologna et al. 2013, Marsili et al. 2019, Holstege 1992, Holstege 2002, Wild et al. 2003, Paradiso et al. 2005). Because these neural systems only partially overlap, WSR symmetry is not expected to track with axial severity. These conceptual differences, together with the limited sample size, likely account for the absence of correlations between WSR symmetry and axial involvement in our cohort.

Another interesting finding is that no differences were found between symmetric and asymmetric PD patients in NMSS, MoCA, BDI, or BAI scores, indicating that neither cognitive nor psychiatric factors contributed to facial symmetry differences. This suggests that neither cognitive status nor psychiatric symptoms, including anxiety, depression, or overall non-motor burden, contribute meaningfully to the degree of facial symmetry during spontaneous smiling. Instead, the pattern we observed appears to reflect a primarily motor phenomenon. This interpretation aligns with prior evidence indicating that hypomimia in PD is largely independent of emotion recognition deficits or cognitive-affective disturbances and is more closely related to bradykinetic motor output itself (Ricciardi et al. 2020, Paparella et al. 2024, Bologna et al. 2016). Furthermore, the lack of association with non-motor domains reinforces the idea that spontaneous facial expressivity, as elicited by the WSR, relies on automatic emotional–motor pathways that may remain relatively preserved even in the presence of cognitive or psychiatric comorbidities.

This study has some limitations. First, the relatively small sample size may have reduced statistical power and contributed to the underestimation of potential associations. Notwithstanding the statistical significance achieved, our findings should be interpreted in the context of the magnitude of the observed effect sizes. As such, caution is required when drawing conclusions, and further replication in larger cohorts will be necessary to establish the consistency and clinical relevance of our results. The analysis addressing concordance between facial laterality and body laterality is particularly limited by the small number of asymmetric cases, which reduces the ability to detect correlations or concordance. The lack of objective quantitative tools, such as automated or landmark-based facial analysis, limited the granularity and precision of the symmetry and expressivity measures. To generate chimeric images, we relied on previous published papers and used the Pichacks web-based tool (Ricciardi et al. 2018, Marsili et al. 2019). This tool originates from an entertainment-oriented website. Our findings could then be reproduced in future studies using standard, verifiable scientific image processing software with more clearly defined geometric procedures. Moreover , hemifacial size asymmetry is a well-documented phenomenon (Koff et al. 1985). We did not apply adjustments for hemifacial size asymmetry, as such corrections may introduce geometric distortions and potentially obscure subtle but meaningful differences in facial motor expression between hemifaces. Future studies will assess the influence of hemifacial size asymmetry on spontaneous smiling. All PD patients were evaluated exclusively in their ON medication state. Although this ensured a standardized clinical condition, it prevents us from determining whether dopaminergic fluctuations modulate spontaneous smile symmetry or the expression of the WSR. Evaluating patients in both ON and OFF states would help clarify the influence of dopaminergic tone on spontaneous facial expressivity. Moreover, testing patients only in their ON medication state may have partially masked appendicular asymmetry, which could in turn influence the observed correlations between facial and body laterality. The cross-sectional design does not allow assessment of how facial symmetry and spontaneous emotional expressivity evolve over time. Longitudinal studies would be valuable to determine whether smile asymmetry changes across disease progression, whether it characterizes early stages of PD, or whether progressive bilateral hypomimia gradually masks physiologically lateralized patterns. We did not examine whether sex influences WSR frequency or asymmetry outcomes. Finally, given the context in which the WSR is elicited, i.e., immediately following a voluntary motor act and within a social/clinical interaction, the resulting smile should not be interpreted as a purely affect-driven spontaneous smile. Rather, WSR likely reflects a composite phenomenon, integrating involuntary motor coupling, emotional aspects, and social modulation. Nonetheless, the smile elicited after whistling remains, at present, the most easily accessible and reproducible proxy for spontaneous smiling in a standardized experimental or clinical setting. Truly affect-driven smiles (e.g., humor-related Duchenne smiles) are considerably more difficult to evoke and study under controlled conditions. We have therefore framed the WSR as a pragmatic surrogate rather than a direct equivalent of emotional smiling. Future studies should directly compare reflex-elicited and affect-driven smiles to assess potential differences in facial characteristics, including symmetry and muscle recruitment, and to refine interpretations regarding the underlying neural mechanisms.

## Conclusion

This study provides novel insights into facial emotional expression in PD. To our knowledge, it represents the first systematic investigation of WSR asymmetry and its relationship with lateralized motor symptoms. Our findings support the use of WSR–based facial analysis and chimeric image paradigms as simple yet sensitive tools for assessing facial hypomimia and asymmetry. Furthermore, they highlight the potential diagnostic value of smile symmetry as a biomarker of disease progression. Future studies using automated facial analysis methods, such as artificial intelligence and machine learning, could enhance the objectivity and accuracy of facial expression quantification and contribute to the development of automated diagnostic and monitoring tools for PD.

## Supplementary Information

Below is the link to the electronic supplementary material.

Supplementary Material 1

## Data Availability

The datasets generated during and/or analyzed during the current study are available from the corresponding author on reasonable request.

## Abbreviations

BAI: Beck Anxiety Inventory
BDI: Beck Depression Inventory
BLI: Body Laterality Index
FLI: Facial Laterality Index
HCs: Healthy Controls
LA: Less Affected (body side)
LEDD: levodopa equivalent daily doses
LL: Left-Left
M1: primary motor cortex
MA: Most Affected (body side)
MoCA: Montreal Cognitive Assessment
MDS-UPDRS: Movement Disorders Society-sponsored version of the Unified Parkinson Disease Rating Scale
NMSS: Non-Motor Symptoms Scale for Parkinson’s Disease
PD: Parkinson’s disease
RR: Right-Right
SD: standard deviation
UPDRS: Sponsored version of the Unified Parkinson Disease Rating Scale
WSR: Whistle–Smile Reflex
