# Supplementary material for: Investigating smiling asymmetries in Parkinson’s disease through the whistle–smile reflex
Source: J Neural Transm (Vienna). 2026 Jul 2;133(9):2535–46. doi: 10.1007/s00702-026-03193-1 (PMC13687223; doi:10.1007/s00702-026-03193-1)
Supplement: Supplementary file 1 [file 702_2026_3193_MOESM1_ESM.docx]

**SUPPLEMENTARY MATERIALS**

**Supplementary Table 1. Demographic and clinical data of patients with Parkinson’s Disease (PD) who smiled after the whistle (smilers) and patients who did not smile (non smilers)**

|  |  | Smilers | Non smilers | p-value |
| --- | --- | --- | --- | --- |
| N° |  | 22 | 28 | / |
| Age |  | 67.2±10.3 | 73.7±8.9 | **0.02** |
| Sex |  | 14 M (40%) | 21 M (60%) | 0.53 |
| Disease Duration |  | 7.5 (5.75) | 4.5 (8.25) | 0.13 |
| Most affected side |  | 12 Right (54.55%) | 14 right (50%) | 0.78 |
| LEDD |  | 630 (484) | 525 (200) | 0.11 |
| MDS-UPDRS part III |  | 24.5 (13) | 26.5 (16.8) | 0.85 |
| NMSS |  | 41.5 (41.3) | 40 (41.3) | 0.75 |
| MOCA |  | 26 (3) | 26.5 (2) | 0.13 |
| BDI |  | 5 (9.5) | 5.5 (7.25) | 0.29 |
| BAI |  | 8 (14.5) | 7.5 (7.5) | 1 |

Age is expressed in years. M: males; MOCA: Montreal Cognitive Assessment; MDS-UPDRS: Movement Disorder Society–sponsored Unified Parkinson’s Disease Rating Scale part III; NMSS: Non-Motor Symptoms Scale for Parkinson’s Disease; BDI: Beck’s Depression Inventory; BAI: Beck Anxiety Inventory. Results are shown as median (interquantile range-IQR) except for age, which resulted normally distributed and is presented as mean values ± 1 standard deviation (SD). p-values from unpaired t test (for age comparison) and from Mann-Whitney U test (and from Fisher’s test for the sex and the more affected side). Significant values are shown in bold.

**Supplementary Table 2. Demographic and clinical data of Parkinson’s Disease (PD) patients and healthy controls (HCs) who smiled after the whistle**

|  |  | PD Smilers | HCs smilers | p-value |
| --- | --- | --- | --- | --- |
| N° |  | 22 | 16 | / |
| Age |  | 67.18±10.29 | 72.06±6.87 | 0.1 |
| Sex |  | 14 (63.64%) | 9 (56.25%) | 0.74 |
| MOCA |  | 26 (3) | 25 (1.5) | 0.42 |
| BDI |  | 5 (9.5) | 0 (6.5) | 0.43 |
| BAI |  | 8 (14.5) | 0 (11.5) | 0.27 |

Age is expressed in years. M: males; MOCA: Montreal Cognitive Assessment; BDI: Beck’s Depression Inventory; BAI: Beck Anxiety Inventory. Results are shown as median (interquantile range-IQR) except for age, which resulted normally distributed and is presented as mean values ± 1 standard deviation (SD). p-values from unpaired t test (for age comparison) and from Mann-Whitney U test (and from Fisher’s test for the sex).
